# Influence of particle parameters on deposition onto healthy and damaged human hair

**DOI:** 10.1111/ics.12994

**Published:** 2024-08-12

**Authors:** Huijun Phoebe Tham, Kah Yuen Yip, Srinivasulu Aitipamula, Srinivasa Reddy Mothe, Wenguang Zhao, Ping Sen Choong, Ayca Altay Benetti, Wanjuan Evonne Gan, Fong Yew Leong, Praveen Thoniyot, Thomas L. Dawson

**Affiliations:** ^1^ A*STAR Skin Research Labs (A*SRL) Agency for Science, Technology and Research (A*STAR) Singapore Singapore; ^2^ Institute of Sustainability for Chemicals, Energy and Environment (ISCE^2^) Agency for Science, Technology and Research (A*STAR) Singapore Singapore; ^3^ Department of Pharmacy National University of Singapore Singapore Singapore; ^4^ School of Biological Sciences Nanyang Technological University Singapore Singapore; ^5^ Institute of High Performance Computing (IHPC) Agency for Science, Technology and Research (A*STAR) Singapore Singapore; ^6^ Department of Chemical Engineering and Chemistry Eindhoven University of Technology Eindhoven The Netherlands; ^7^ Center for Cell Death, Injury Regeneration, Departments of Drug Discovery Biomedical Sciences and Biochemistry Molecular Biology Medical University of South Carolina Charleston South Carolina USA; ^8^ A*STAR Skin Research Labs (A*SRL) Agency for Science, Technology and Research (A*STAR) & Skin Research Institute of Singapore (SRIS) Singapore Singapore

**Keywords:** delivery, deposition, formulation/stability, hair treatment, kinetics, nanoparticles

## Abstract

**Objective:**

This research investigates how particle parameters, such as zeta potential, size, functional group, material composition, and hydrophobicity affect their affinity and deposition of particles onto hair.

**Methods:**

Streaming potential was used as the technique for analysis. The streaming potential data obtained was then converted to surface coverage data. Scanning electron microscopy (SEM) was also done to visualize particle localization on the hair surface.

**Results:**

This study found stronger particle affinity on healthy than on damaged (oxidatively bleached) hair, due to diminished interaction sites from the removal of the hair shaft's external lipid layer. SEM imaging supported these findings and offered insights into particle localization. Hydrophilic silica particles accumulated along the exposed hydrophilic cuticle edges of healthy hair, due to hydrogen bonding with the exposed endocuticle. This localization is hypothesized to be due to the limited hydrophilic binding sites on the hydrophobic healthy hair cuticle surface. In damaged hair, an abundance of hydrophilic sites across the cuticle surface results in more dispersed binding. Hydrogen bonding and electrostatic attraction were shown to be the predominant forces influencing deposition, with hydrophobic interactions playing a less influential role. The affinity studies also proved that electrostatic attractions work over a longer range and are more effective at lower particle conditions compared with hydrogen bonding which only start to play a bigger role at higher particle concentrations. Steric hindrance of bulky side groups acted as a significant repulsive force. Results also revealed that larger particles deposit poorly on both healthy and damaged hair compared with smaller ones. Compared with neutrally charged silica nanoparticles (SN‐2), positively charged PMMA particles (PN+16) have a stronger affinity to healthy hair, with highly charged particles (PN+49) depositing most rapidly.

**Conclusion:**

This study provides a fundamental understanding of how particle–surface parameters influence their affinity to hair and how damaging hair affects deposition.

## Graphical abstract

This study utilized streaming potential to explore particle affinity to hair, considering factors, such as zeta potential, size, and hydrophobicity. Smaller particles deposited better than bigger particles. Neutrally charged particles were found to deposit best on both healthy and damaged hair while positively charged particles deposited the fastest at low concentrations.

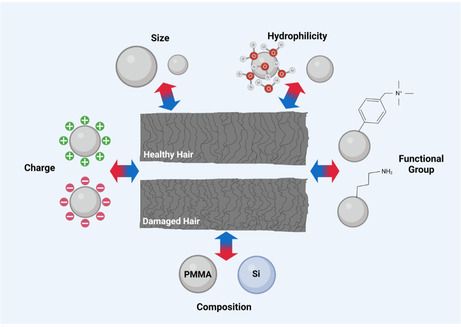

## Résumé

**Objectif:**

Cette étude examine comment les paramètres des particules, tels que le potentiel zêta, la taille, le groupe fonctionnel, la composition du matériau et l’hydrophobie, affectent l’affinité et le dépôt des particules sur les cheveux.

**Méthodes:**

Le potentiel d’écoulement a été utilisé comme technique d’analyse. Les données de potentiel d’écoulement obtenues ont ensuite été converties en données de couverture de surface. Une microscopie électronique à balayage (MEB) a également été réalisée pour visualiser la localisation des particules à la surface des cheveux.

**Résultats:**

Cette étude a mis en évidence une affinité plus forte des particules sur des cheveux sains que sur des cheveux abîmés (décolorés par oxydation), en raison de la diminution des sites d’interaction due à l’élimination de la couche lipidique externe de la tige du cheveu. L’imagerie MEB a confirmé ces résultats et a permis d’obtenir des informations sur la localisation des particules. Des particules de silice hydrophile se sont accumulées en bordure des cuticules hydrophiles exposées des cheveux sains, en raison de la liaison de l’hydrogène avec l’endocuticule exposée. Il est supposé que cette localisation est due au nombre réduit de sites de liaison hydrophiles à la surface hydrophobe saine de la cuticule capillaire. Sur des cheveux abîmés, l’abondance de sites hydrophiles sur la surface des cuticules entraîne une liaison plus dispersée. La liaison de l’hydrogène et l’attraction électrostatique se sont avérées être les forces prédominantes qui influencent le dépôt, les interactions hydrophobes jouant un rôle moins influent. Les études d’affinité ont également démontré que les attractions électrostatiques fonctionnent sur une plus longue plage et sont plus efficaces dans des conditions de concentration de particules inférieures par rapport à la liaison de l’hydrogène qui ne commence à jouer un rôle plus important qu’à des concentrations de particules plus élevées. L’entrave stérique des groupes latéraux volumineux a agi comme une force répulsive significative. Les résultats ont également révélé que les particules plus grosses se déposent faiblement sur des cheveux sains et des cheveux abîmés par rapport aux particules plus petites. Par rapport aux nanoparticules de silice à charge neutre (SN‐2), les particules de PMMA à charge positive (PN+16) ont une affinité plus forte avec les cheveux sains, les particules fortement chargées (PN+49) se déposant le plus rapidement.

**Conclusion:**

Cette étude apporte une compréhension fondamentale de la façon dont les paramètres de la surface et des particules influencent leur affinité avec les cheveux et dans quelle mesure les cheveux abîmés affectent les dépôts.

## INTRODUCTION

Hair serves as a crucial aspect of personal appearance and is therefore a subject of broad interest. There are a wide variety of hair care products, for example, shampoos, conditioners, hair serums, styling products, such as hair wax, hair spray, volumizing products, and colourants, etc. Many have already incorporated nanoparticulate or microparticulate‐based formulations [1]. However, most are protected and undisclosed to the public. Nanoparticles and microparticles are used in hair product formulations for a multitude of benefits, such as (i) enhancing hair's optical or physical appearance; (ii) enhancing the efficacy while reducing the irritation or toxicity of active compounds; (iii) controlling compound release; and (iv) enhancing the targeted delivery of active compounds into the hair follicle or onto the shaft [2, 3]. The efficacy of hair products in delivering active ingredients to the hair surface or improving hair physical properties depends on the ability of particles to deposit effectively onto hair strands. Understanding the influence of particle parameters on deposition can significantly impact the formulation and development of innovative hair care products.

There have been several reports of depositing nanomaterials to hair. For example, halloysite clay nanotubes have been coated on hair for photoprotective applications [4], and diatomite encapsulated silver nanoparticles have been reported to be viable hair dyes [5]. Poly‐γ‐glutamic acid/glycol chitosan nanoparticles have also been used to incorporate p‐phenylenediamine for reduced scalp irritation during hair dyeing [6]. Silicone oil nanoemulsions have also been developed for conditioning applications [7] while zinc oxide nanoparticles have been coated with ketoconazole for anti‐dandruff applications [8]. Techniques such as high‐performance liquid chromatography (HPLC) [9] or fluorescence intensity [10] have traditionally been used to quantify the amount of product deposition on hair but quantifying particle deposition has posed a challenge due to the complex fibre nature, making it difficult to be simply modelled. Mass and numerical balance methods such as single‐particle optical sensing (SPOS) and nanoparticle tracking analysis (NTA) are not sufficiently accurate due to the lack of commercially available standards and the high signal‐to‐noise ratio due to the low deposition on hair. Analysis methods such as scanning electron microscopy (SEM) and atomic force microscopy (AFM) have also been inefficient due to the small scale of measurement, with images often unrepresentative enough for accurate quantification. The top‐down two‐dimensional imaging approach also renders this method of analysis challenging. Furthermore, the deposition of hair products on damaged hair has long been a challenge for formulation scientists. Damaged hair often encompasses a loss of essential lipids and proteins, resulting in decreased surface hydrophobicity and weakened intermolecular interactions [11]. Consequently, damaged hair has weaker binding forces with hair products, leading to poor adhesion [12], limiting the hair product's ability to function effectively.

**FIGURE 1:**
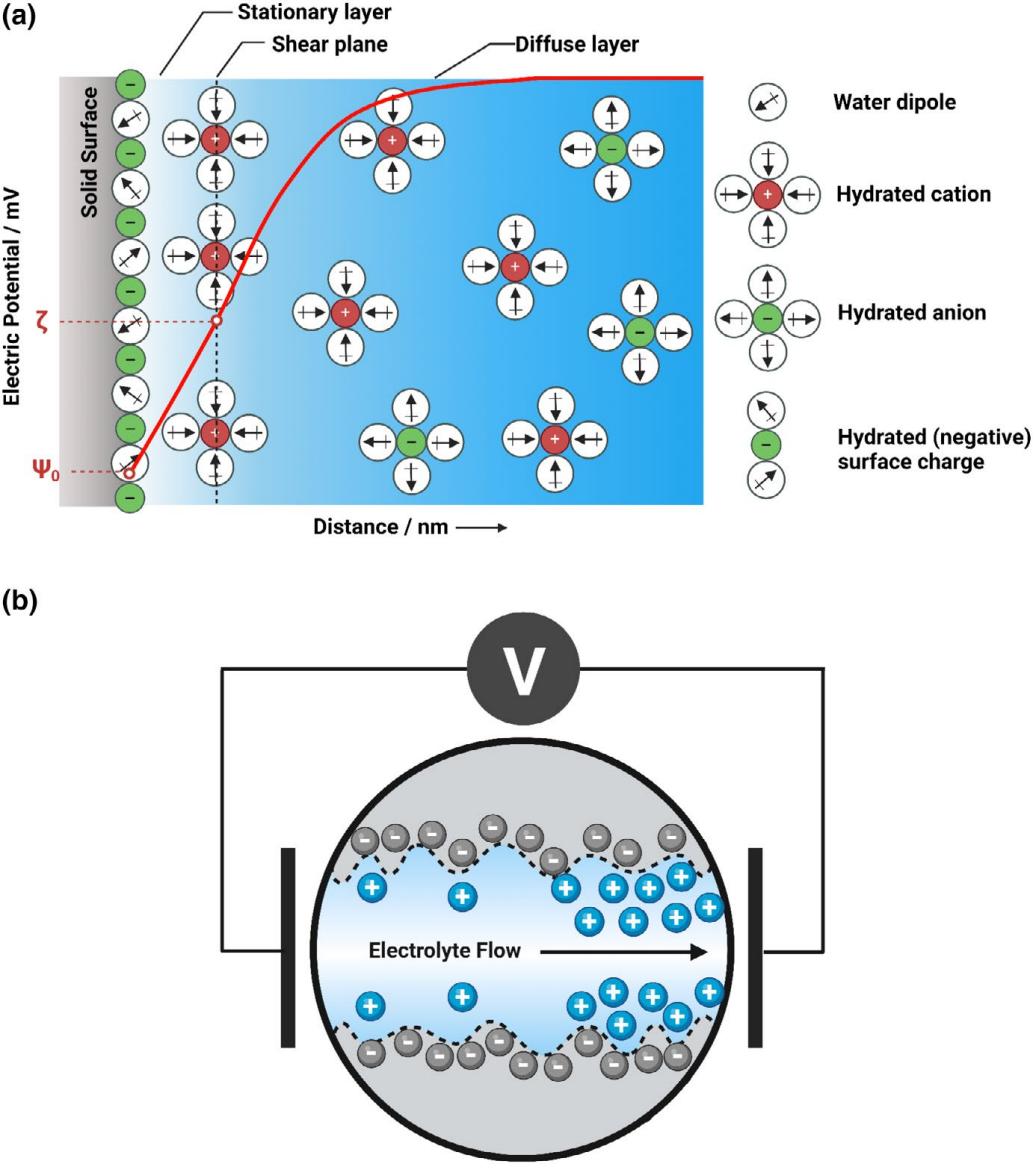
(a) Model of the electrochemical double layer at the solid–liquid interface (Ψ_0_: surface potential, ζ: zeta potential). (b) Schematics of the streaming potential technique, illustrating electrolyte flow in a capillary channel and the resulting charge separation. Figures adapted from [13]. [Colour figure can be viewed at wileyonlinelibrary.com]

To quantitatively evaluate particle deposition on hair, this study utilized electrokinetic analysis. Electrokinetic analysis quantifies the electrophysical characteristics and actions of charged particles or surfaces. It measures interactions between electric fields and charged particles or surfaces to extract valuable insights into interfacial properties, such as zeta (ζ) potential. Zeta potential is defined as the electrical potential at the boundary layer between the Stern layer and the diffuse layer, as illustrated in Figure 1a. This property of solid surfaces provides information about the surface charge, functional groups, electrostatic attraction or repulsion, as well as specific adsorption of particulates or compounds with the solid surface. Streaming potential, defined as the electrokinetic potential which arises when an electrolyte solution interacts with a charged surface, allows for real‐time adsorption and desorption studies [13]. This technique provides results that reflect the properties of an entire hair strand or a bundle of hair strands, in contrast to SEM or AFM, which offer only localized insights. Unlike mass balance, this method only considers the surface of the hair and not particles that could have entered the hair shaft, hence a better measure of particle‐hair affinity.

Zeta potential can be assessed through various techniques, including electrophoresis, electroosmosis, streaming potential, and sedimentary potential. However, for solid surfaces like hair fibres, the preferred analytical method is streaming potential. Streaming potential is created by either flowing a liquid tangentially to a flat solid surface, or as employed in this study, by forcing the liquid through a permeable sample such as a fibre plug. A pressure gradient is applied to the ends of the system, and acts as the driving force for the liquid to flow, generating a streaming potential [14], as illustrated in Figure 1b. By measuring the gradient of streaming potential with differential pressure, zeta potential can be calculated using the modified Helmholtz‐Smoluchowski equation:

(1) $$\zeta =\frac{{dU}_{\mathrm{str}}}{\mathrm{d\Delta}p}\times \frac{\eta }{\varepsilon \times \varepsilon_{o}}\times \kappa_{B}$$

where ζ is zeta potential, *U*_str_ is streaming potential, Δ*p* is differential pressure, *η* is the viscosity of the electrolyte solution, *ε* is the dielectric strength of the electrolyte solution, *ε*_o_ is the relative permittivity of vacuum, and *κ*_*B*_ is the conductivity of the electrolyte solution.

Several particle–surface forces potentially occur. Briefly, (i) electrostatic (Coulombic) interactions arise from the attraction of oppositely charged surfaces and (ii) van der Waals forces occur due to fluctuations in electron distribution within particles and surfaces. Though weaker than electrostatic attractions, van der Waals forces play a crucial role through the cumulative effects of attraction. (iii) Hydrogen bonding arises when hydrogen atoms covalently bonded to highly electronegative atoms interact with other electronegative atoms. (iv) Hydration/solvation forces occur when water/solvent molecules arrange themselves around solute particles or surfaces in structured hydration/solvation shells. Interactions between these shells can result in hydration/solvation forces that either attract or repel particles and surfaces. (v) Steric interactions arise from the repulsion between particles or surfaces because of the excluded volume principle whereby particles or molecules cannot occupy the same space simultaneously.

We report a technique to quantify particle deposition on hair with an aim to explore the relationship between particle characteristics, such as size, charge, and functional groups, with their ability to deposit onto healthy and bleached human hair. The influence of formulation on deposition was not considered in this work, and to the best of our knowledge, this is among the first studies to investigate the fundamentals of particle deposition to hair.

## MATERIALS AND METHODS

### Hair tresses

Blended hair tresses were obtained from International Hair Importers & Products (IHIP, USA). Healthy hair was standard REMY hair reported to be free from chemical treatment and damaged hair was taken from the same batch of healthy hair and subsequently bleached with hydrogen peroxide using a standardized in‐house protocol by IHIP. All hair samples were cleaned with 1% sodium dodecyl sulphate (SDS) and well‐rinsed before use. Hair was weighed and bundled into 1 g tresses with no further treatment. Hair tresses were inspected by SEM to ensure that the cuticles were only chemically damaged, but physically intact with no exposed cortex and minimum lifted cuticles. This was done to focus on studying the influence of chemical damage on particle adhesion, without any interference from the physical entrapment of particles between the cuticles.

### Scanning electron microscopy (SEM)

JEOL JSM‐6701F FEG SEM was used for hair sample imaging. Hair samples were mounted on a sample holder and coated with 4 nm platinum in a sputter coater (LEICA EM ACE200) before imaging, and before loading into the SEM machine. Particle images were provided by the Institute of Sustainability for Chemicals, Energy and Environment (ISCE^2^), A*STAR using a JEOL JSM‐7900F FEG SEM.

### Dynamic light scattering (DLS)

Dynamic light scattering (DLS) was conducted on a Malvern Zetasizer Nanoseries at 25°C. 20 μL sample was added into a thin‐wall polystyrene cuvette and then diluted with 2 mL ultrapure water before analysis.

### Degree of wetting measurements

Contact angle and wetting tension measurements were carried out using a FTA200 instrument with FTA32 software. Particles were purified and carefully dropcast onto a microscope glass slide and dried overnight.

### Electrokinetic analysis

A solid‐state electrokinetic analyser (SurPASS 3; Anton Paar GmbH, Austria) with a cylindrical cell mounted, was used to measure the streaming potential of hair samples.

### Adsorption kinetics

1 mM potassium chloride (KCl) solution was used as the electrolyte. The pH was adjusted to 7 before analysis. Hair samples were pre‐soaked in the electrolyte solution for 15 minutes to equilibrate prior to analysis. Particles were centrifuged and rinsed with ultrapure water to remove any surfactants or stabilizers used in their synthesis. A stable baseline was first obtained before particles were added into the electrolyte reservoir to correspond to a final concentration of either 0.0005%, 0.0025%, or 0.01% w/v. These concentrations were chosen due to their relevance to consumer hair products. It should be noted that using the same %w/v concentration would result in a lower number of particles of PM+16 per volume compared with the nanoparticles. This could affect the surface coverage of PM+16 on the hair. The streaming potential was measured for 2400 s after particle addition due to instrument limitations, and care was taken to keep the pH within ±0.2 points. Experiments were carried out in duplicates, and the data for each run were binned and averaged.

The deposition efficacy of the particles was analysed by measuring the proportion of hair surface covered by particles, *θ*(%). This was derived by first calculating zeta potential (*ζ*) data from streaming potential data output using the variation of the Helmholtz‐Smoluchowski equation (Equation 1). After which, the surface coverage (*θ*) was obtained by solving the following equation obtained from Ekiel‐Jezewska et al. [15] :

(2) $$\frac{\zeta \left(t\right)}{\zeta_{P}}=\left(\frac{\zeta_{I}}{\zeta_{P}}-b\right)e^{-c_{I}\theta \left(t\right)}+\mathrm{a\theta}\left(t\right)+b$$

where *c*_*I*_ = 10.2037, a virial coefficient, *a* = 0.202, a fitting parameter, *b* = (*c*_P_ − *a*)/*c*_*I*_ ≈ 0.6182, $\zeta_{I}$ is the starting zeta potential of the interface (hair), $\zeta_{p}$ is the zeta potential of particle, and $\zeta \left(t\right)$ is the zeta potential of the interface (hair) as a function of time.

### Fitting

For overall fitting from 0 to 2400 s, a modified logarithmic function was used:

(3) $$f\left(x\right)=y_{0}+a∙\ln\left(b∙\left(x-x_{0}\right)+1\right)$$

where *f*(*x*) is the fitted variable, *a*, *b*, *x*_0_ and *y*_0_ are the fitting parameters, and *x* is the independent variable.

For fitting from 0 to 60 s, a second‐degree polynomial fitting function was used:

(4) $$f\left(x\right)=ax^{2}+\mathrm{bx}+c$$

where *f*(*x*) is the fitted variable, *x* is the independent variable, and *a*, *b* and *c* are fitting coefficients estimated during the fitting process.

### Data analysis

All data analysis (binning, equation solving, and curve fitting) were carried out using Python code.

### Particles

**FIGURE 2:**
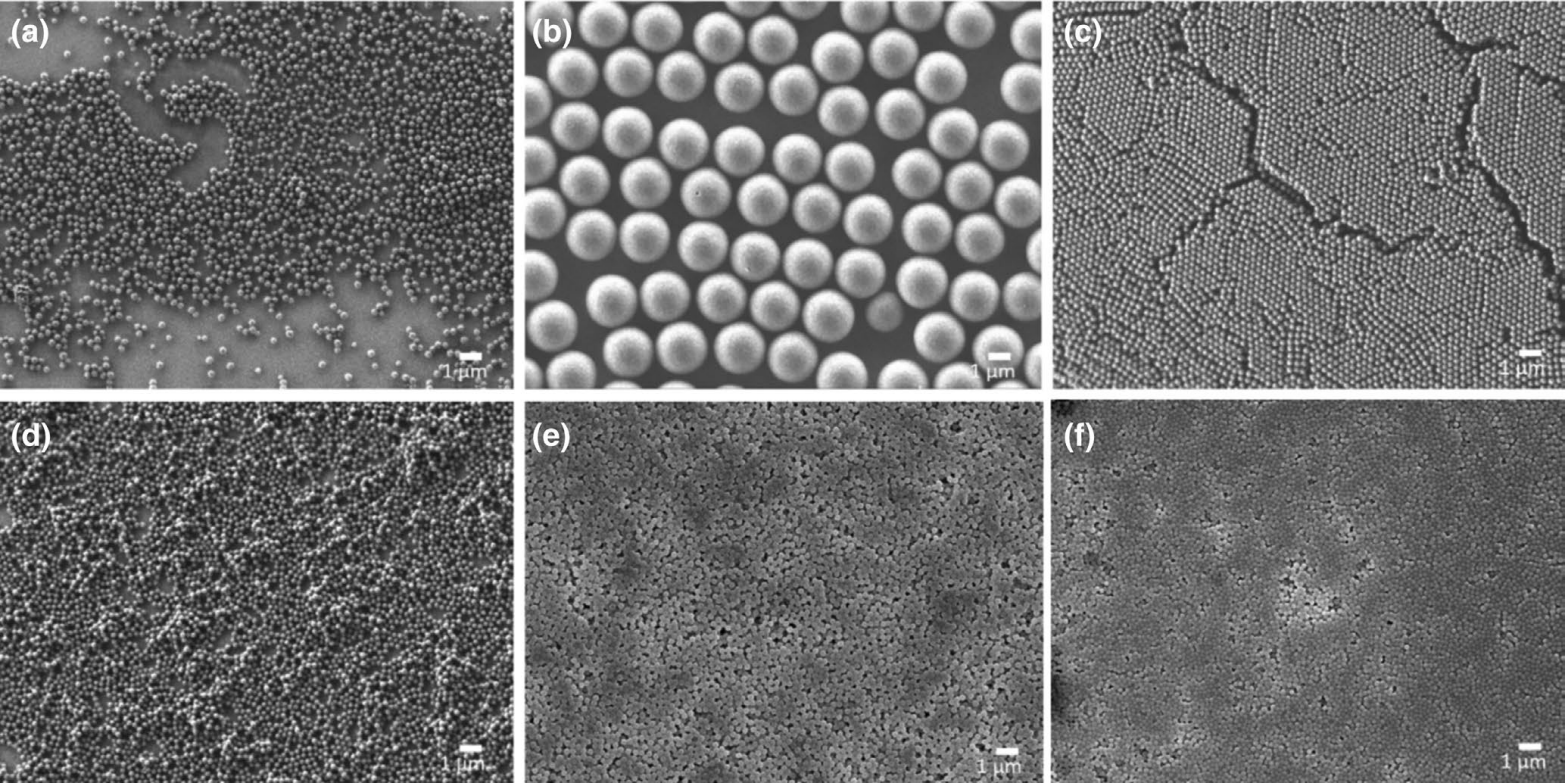
SEM images of (a) PN+16, (b) PM+16, (c) PN‐40, (d) PN+49, (e) SN‐2 and (f) SN‐46.

In this research study, we examined spherical particles composed of either silica or PMMA‐based materials. The particles were named as follows: PM+16, PN+16, PN‐40, PN+49, SN−2 and SN−46. Here, ‘P' represents PMMA‐based particles, ‘S' represents silica‐based particles, ‘M' denotes micron‐sized particles, and ‘N' denotes nano‐sized particles. The numerical values represent the zeta potential of each particle. Their hydrodynamic diameter, zeta potential, and degree of wetting are summarized in Table 1 below and corresponding electron microscope images are shown in Figure 2.

**TABLE 1:** Particles used for hair adsorption experiments.

Particle	Composition	Size (nm)	Zeta potential (mV)	Contact angle (^o^)	Wetting tension (mM/mm)
PM+16	MMA, AEMA	2540	+16	38.7	56.9
PN+16	MMA, AEMA	370	+16	72.1	20.4
PN‐40	MMA	363	−40	50.9	46.0
PN+49	MMA, VBTMA	293	+49	63.3	32.4
SN‐2	TEOS, APTES	432	−2	19.9	68.6
SN‐46	TEOS	282	−46	7.1	72.3

*Note*: Zeta potential was measured at pH 7 in 1 mM KCl solution.

Abbreviations: AEMA, 2‐aminoethylmethacrylamide hydrochloride; APTES, (3‐aminopropyl) triethoxysilane; MMA, methyl methacrylate; TEOS, tetraethyl orthosilicate; VBTMA, vinylbenzyl trimethylammonium.

## RESULTS

### Degree of wetting

SN‐46 had the lowest contact angle and highest wetting tension, indicating that it had the highest hydrophilicity, which was typical of silica particles. It is followed by SN‐2, PM+16, PN‐40, PN+49, and then PN+16, in increasing order of degree of wetting. Even though PN+16 and PM+16 were synthesized using the same method and had the same functional groups and zeta potential, their contact angles were very different (38.7° for PM+16 and 72.1° for PN+16). This discrepancy can likely be attributed to the sample preparation method, where particles were dropcast to create a film. Smaller particles tend to create a rougher surface compared with larger particles, which can result in a larger contact angle, as the liquid partially occupies the pockets formed by the roughness, referred to as the Wenzel state [16]. In such cases, the contact angle may not accurately reflect the true hydrophobicity and could yield inflated values. Thus, the contact angle should be taken as a rough gauge of hydrophobicity, especially for films made of different particle sizes.

### Adsorption studies

**FIGURE 3:**
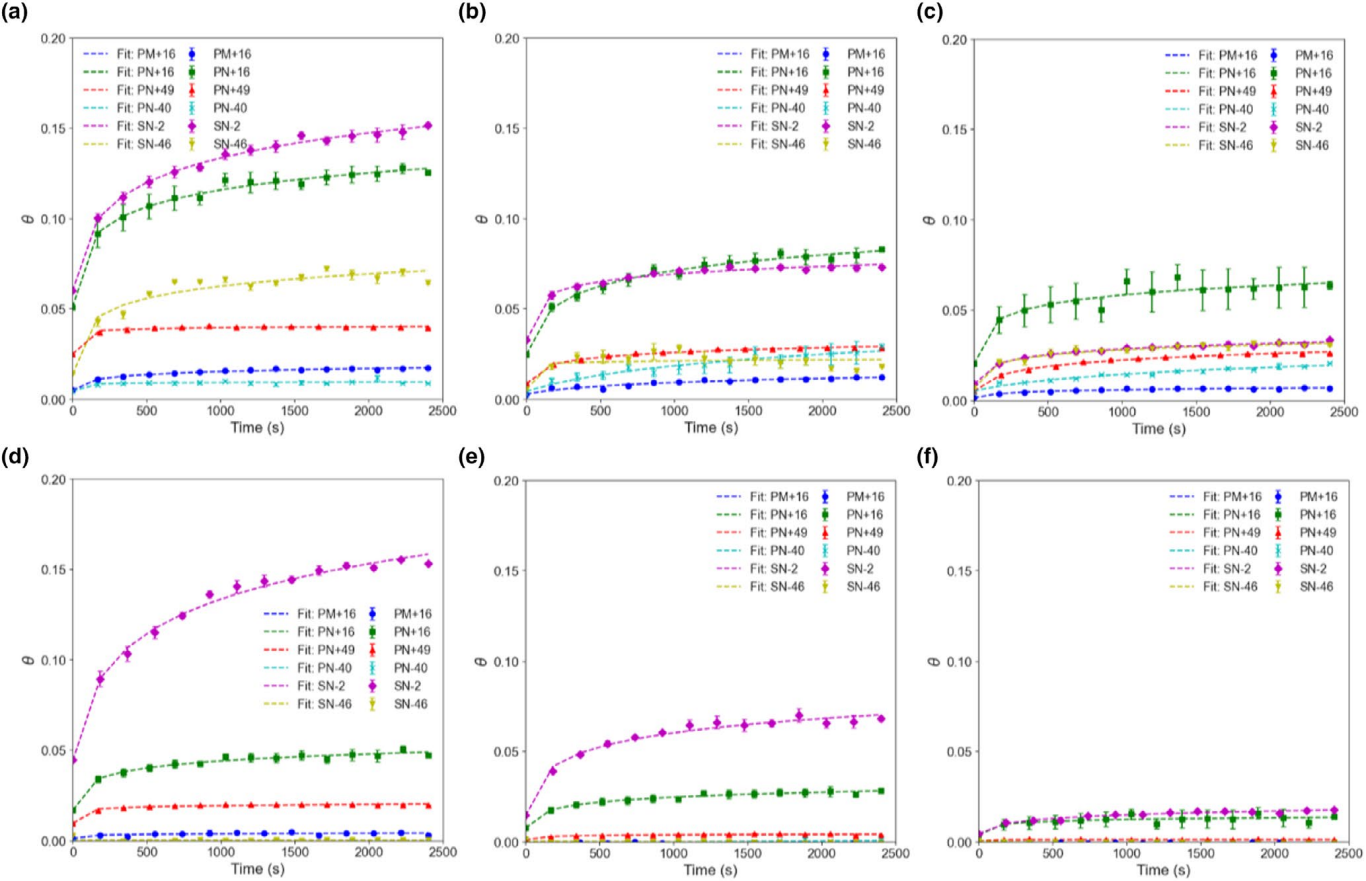
Surface coverage (*θ*) as a function of time for healthy hair with (a) 0.01%, (b) 0.0025%, (c) 0.0005% w/v particle concentration added, and for damaged hair with (d) 0.01%, (e) 0.0025%, and (f) 0.0005% w/v particle concentration added, *n* = 2. Error bars = standard error of the mean. Curves were fitted using a modified logarithmic fitting function. [Colour figure can be viewed at wileyonlinelibrary.com]

Baseline zeta potentials of the healthy and damaged hair tresses were measured to be −36.8 ± 2.2 mV and −5.4 ± 1.7 mV respectively (*n* = 25 each). A concentration‐dependent adsorption study was conducted to measure the deposition efficiency at equilibrium. The changes in surface coverage (*θ*) with time for the particles were plotted in Figure 3. Particles were dispersed in ultrapure water to obtain three different concentrations (0.0005%, 0.0025%, and 0.01% w/v). The baseline noise level was established at 4%, and any deposition below this threshold was not initially regarded as significant. Nevertheless, for the purposes of this study, data points below the 4% threshold were included in the analysis. However, it is advisable to interpret these data points with caution. As the particle concentration was fixed using %w/v units, this meant that there would be less particles per volume for PM+16 compared with the other particles, which could affect PM+16's surface coverage on hair. Generally, all particles followed a concentration‐dependent deposition, with *θ*_0.01%_ > *θ*_0.0025%_ > *θ*_0.0005%_ except for PN‐40 and SN‐46. However, this deviation could be attributed to their coverage being below the 4% threshold level. For all particles, more deposition was observed on healthy hair than on damaged hair, except for SN‐2, which had similar deposition on both healthy and damaged hair. SN‐2, PN+16 and PN+49 were the only three particles which could deposit on damaged hair. Chemically damaged hair has oxidized groups such as sulfonic groups on its surface and is known to be more hydrophilic, porous, and absorbs water more than healthy hair [17], with a lower amount of lipid layer [11]. On the other hand, a healthy hair surface is known to be more hydrophobic as it is rich in polar functional groups, such as amines, thiol, carboxyl, hydroxyl groups, and lipid layers, such as ceramides, fatty acids, and cholesterol [18]. The lower particle deposition on damaged hair was most likely due to the loss of surface lipids and fatty acids during bleaching [11], leading to lower availability of binding sites for interaction with particles [19].

PM+16: From Figure 3, microparticle PM+16 had low deposition across all concentrations for both healthy and damaged hair, depositing much less than the smaller‐sized particles irrespective of the concentration used. Even when compared with PN+16, which shared the same zeta potential of +16 mV, PM+16's deposition on healthy hair at the highest particle concentration (0.01%) reached only 1.7 ± 0.004% coverage, while PN+16 achieved significantly higher coverage at 12.5 ± 0.07%. PM+16's deposition was also lower than SN‐2 and SN‐46, both possessing a higher degree of wetting and carrying negative charges which were expected to deposit the least. This proves that microparticles tend to deposit less effectively than nanoparticles. This could be attributed to PM+16's low surface area to volume ratio, reducing hydration forces and hence reduced attraction. In contrast to smaller particles, bigger particles are less influenced by Brownian motion [20], reducing their interaction with hair and hence lower probability of deposition.

SN‐2: SN‐2 had one of the highest affinities among all particles at 15.2 ± 0.1% coverage on healthy hair at the highest particle concentration. This could be attributed to its tendency to form hydrogen bonds and lack of electrostatic repulsion. SN‐2 also deposited equally well on damaged hair at 15.3 ± 0.04% coverage. Its higher hydrophobicity relative to SN‐46 may have played a role in its adhesion to the hair surface through hydrophobic interactions. The comparable zeta potential between damaged hair and SN‐2 appeared to enhance their mutual interaction as well. This behaviour aligns with findings from previous studies involving like‐charged colloidal particles, where phenomena such as charge fluctuations [21] or interfacial solvation forces [22] gave rise to long‐range attractive forces. It is plausible that this attraction theory could be extended to particle–surface interactions too. Furthermore, at near‐neutral zeta potentials, electrostatic charges are minimal and there is high tendency for interaction between surfaces.

PN‐40: PN‐40 displayed low deposition across all concentrations for both types of hair, achieving only 0.9 ± 0.01% coverage at the highest particle concentration on healthy hair, and negligible attraction to damaged hair. This was most likely due to the electrostatic repulsion between PN‐40 and the negatively charged hair surface. Its high magnitude of zeta potential also gave it colloidal stability, with a decreased tendency to interact with other surfaces.

PN+49: Despite PN+49 having a significantly high and opposite charge to the hair surface and having a relatively low hydrophilicity with a contact angle of 63.3°, its deposition levels remained rather low at only 3.9 ± 0.0009% deposition on healthy hair and 1.9 ± 0.002% on damaged hair at the highest particle concentration. The limited coverage observed could be due to the presence of bulky VBTMA groups on the surface, contributing to steric hindrance and impeding deposition onto the hair surface. Additionally, in an aqueous environment, particles and hair are both surrounded by water molecules, resulting in charge screening, whereby there is a reduction in attraction between oppositely charged entities, diminishing their effective interaction [23].

SN‐46: Even though SN‐46 had a strongly negative zeta potential, it deposited relatively well on healthy hair (6.5 ± 0.09%) but negligibly on damaged hair. The deposition on healthy hair was much higher than similarly charged PN‐40 (0.9%). SN‐46's higher hydrophilicity than PN‐40 meant a lower tendency for hydrophobic interactions to occur with hair, which then suggests that hydrogen bonds exert a more significant influence on deposition compared with hydrophobic interactions. This is also supported by the fact that hydroxyl groups on the surface of silica are known to form stronger hydrogen bonds than the ester groups on the surface of PMMA due to their more electronegative oxygen. But, like PN‐40, the high negative charge on SN‐46 likely resulted in electrostatic repulsion with the hair surface, preventing higher deposition. SN‐46 also deposited poorly on damaged hair despite its ability to form hydrogen bonds. This was also most likely due to the reduced binding sites on damaged hair, exacerbated by the electrostatic repulsion effect, further hindering deposition.

The surface coverages of the particles to hair were tabulated and ranked in Table 2 below. The data for PM+16, PN‐40, and SN‐46 on damaged hair were excluded from the analysis as they showed no appreciable deposition. The weighted average was calculated by taking (0.01% * *θ*_0.01%_ + 0.0025% * *θ*_0.0025%_ + 0.0005% * *θ*_0.01%_)/0.0013% for each particle on both healthy and damaged hair. For healthy hair, the weighted average of *θ*_SN‐2_ > *θ*_PN+16_ > *θ*_SN‐46_ > *θ*_PN+49_ > *θ*PM+_16_ > *θ*_PN‐40_, and for damaged hair, *θ*_SN‐2_ > *θ*_PN+16_ > *θ*_PN+49_. Due to the nature of binning, it would be inaccurate to establish significance.

**TABLE 2:** Summary of surface coverages (*θ*) obtained, average ± standard error of the mean.

Particle	Healthy			Damaged			Weighted average	
	0.01%	0.0025%	0.0005%	0.01%	0.0025%	0.0005%	Healthy	Damaged
PM+16	1.7 ± 0.004%	1.2 ± 0.02%	0.7 ± 0.01%	–	–	–	1.6 ± 0.001%	–
PN+16	12.5 ± 0.07%	8.3 ± 0.08%	6.4 ± 0.2%	4.7 ± 0.001%	2.8 ± 0.001%	1.4 ± 0.001%	11.5 ± 0.006%	4.2 ± 0.004%
PN‐40	0.9 ± 0.01%	2.9 ± 0.1%	2.0 ± 0.002%	–	–	–	1.33 ± 0.004%	–
PN+49	3.9 ± 0.001%	2.8 ± 0.03%	2.6 ± 0.003%	1.9 ± 0.002%	0.4 ± 0.001%	0.1 ± 0.001%	3.6 ± 0.001%	1.5 ± 0.001%
SN‐2	15.2 ± 0.1%	7.3 ± 0.04%	3.3 ± 0.03%	15.3 ± 0.04%	6.8 ± 0.1%	1.8 ± 0.01%	13.2 ± 0.006%	13.2 ± 0.003%
SN‐46	6.5 ± 0.09%	1.8 ± 0.04%	3.0 ± 0.03%	–	–	–	5.5 ± 0.005%	–

**FIGURE 4:**
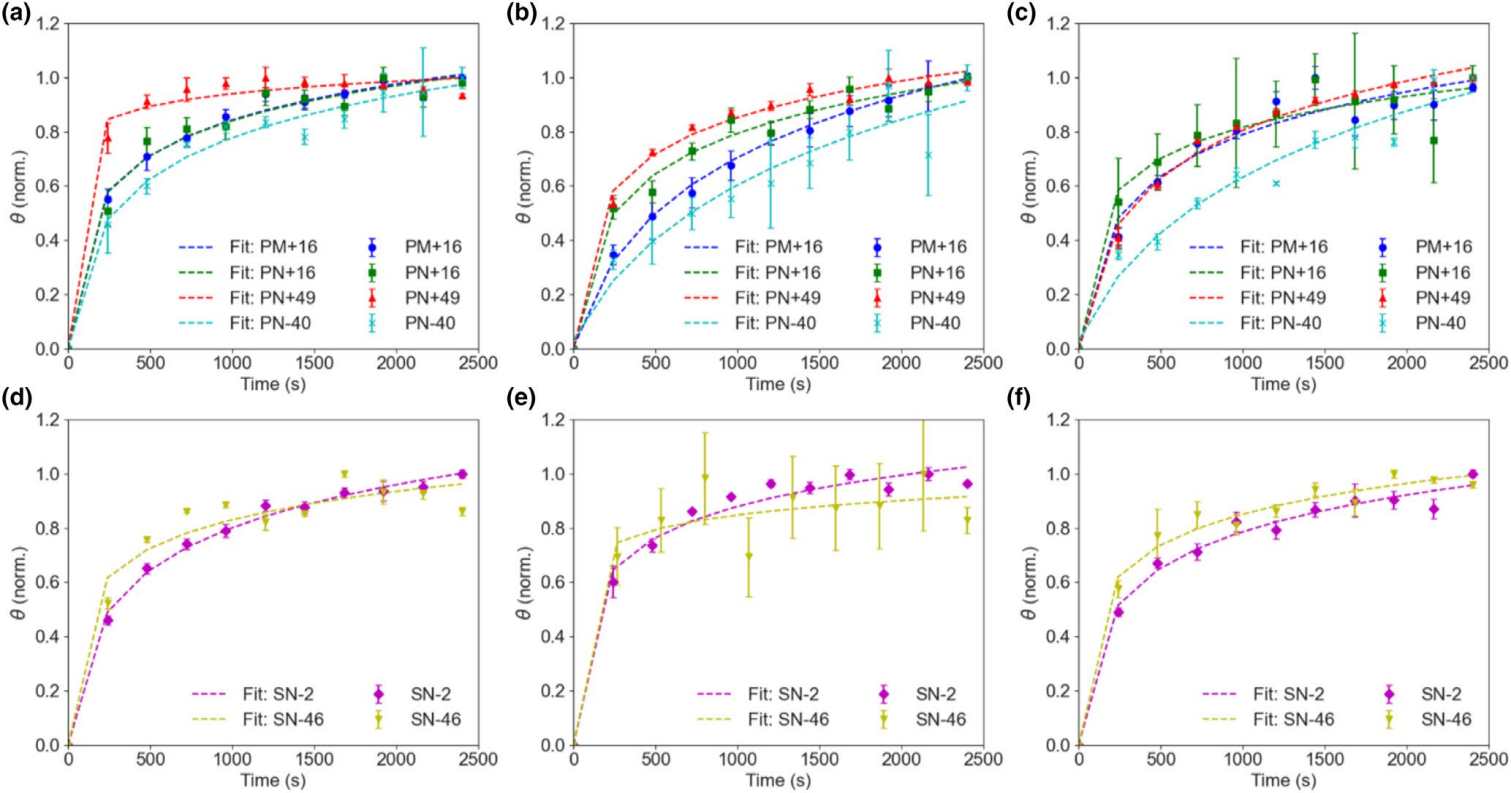
Normalized surface coverage (*θ*) over 2400 s for healthy hair with (a, d) 0.01%, (b, e) 0.0025%, (c, f) 0.0005% w/v particle concentration added. Fitting curves are a guide. [Colour figure can be viewed at wileyonlinelibrary.com]

**FIGURE 5:**
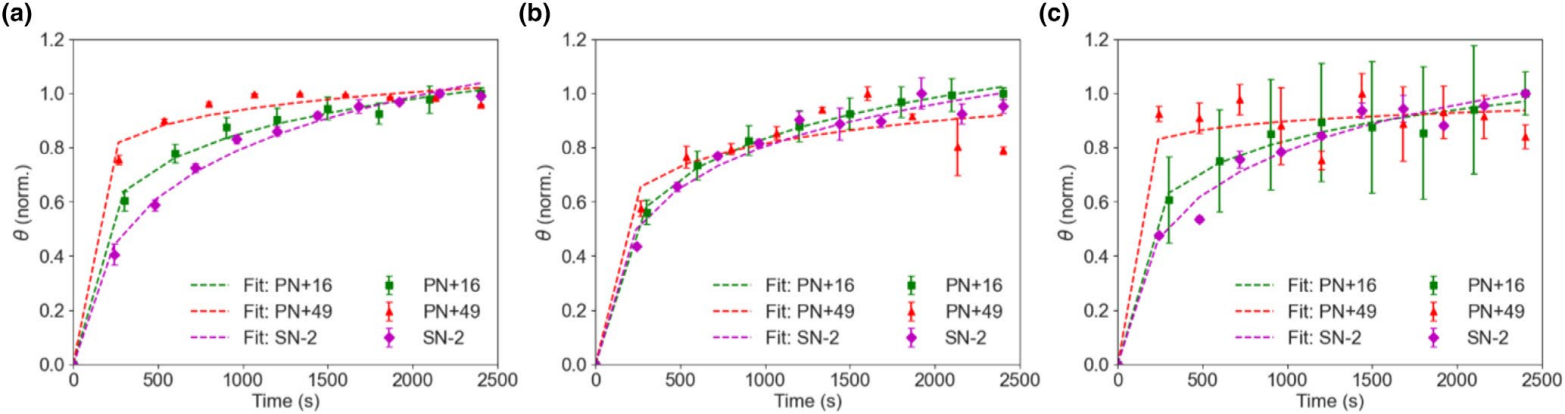
Normalized surface coverage (*θ*) over 2400 s for damaged hair with (a) 0.01%, (b) 0.0025% and (c) 0.0005% w/v particle concentration added. Fitting curves serve as a guide. [Colour figure can be viewed at wileyonlinelibrary.com]

The surface coverage data in Figure 3 were normalized to understand the overall kinetics of the deposition (Figures 4 and 5) and data points were fitted using a modified logarithmic equation (Equation 3). It was apparent that PN+49 had the greatest affinity to both healthy and damaged hair, reaching almost equilibrium as quickly as within 1200 s at the highest particle concentration (Figure 4a, 5a). Its initial gradient measured in the first 150 s was also the steepest among all the polymeric particles for the two highest concentrations (Table S1). At lower particle concentrations, however, the rate at which PN+49 took to deposit decreased. The other particles deposited at a slower rate and still did not reach equilibrium even at 0.01% concentration, suggesting that their reported equilibrium surface coverages were underestimated. PN‐40 deposited the slowest on healthy hair, as it had the gentlest gradient across all concentrations (Table S1). Surprisingly, SN‐46 deposited relatively quickly, with a gradient even higher than PN+16 and SN‐2, however, the rate of deposition was relatively constant across all concentrations, which confirms that its deposition rate is not concentration‐dependent. Likewise, SN‐2 and PN+16 on healthy hair, as well as all three particles on damaged hair also did not show a clear concentration‐dependent behaviour with similar deposition rates across all particle concentrations. PN+49 was the only particle which displayed a clear concentration‐dependent deposition rate on healthy hair.

**FIGURE 6:**
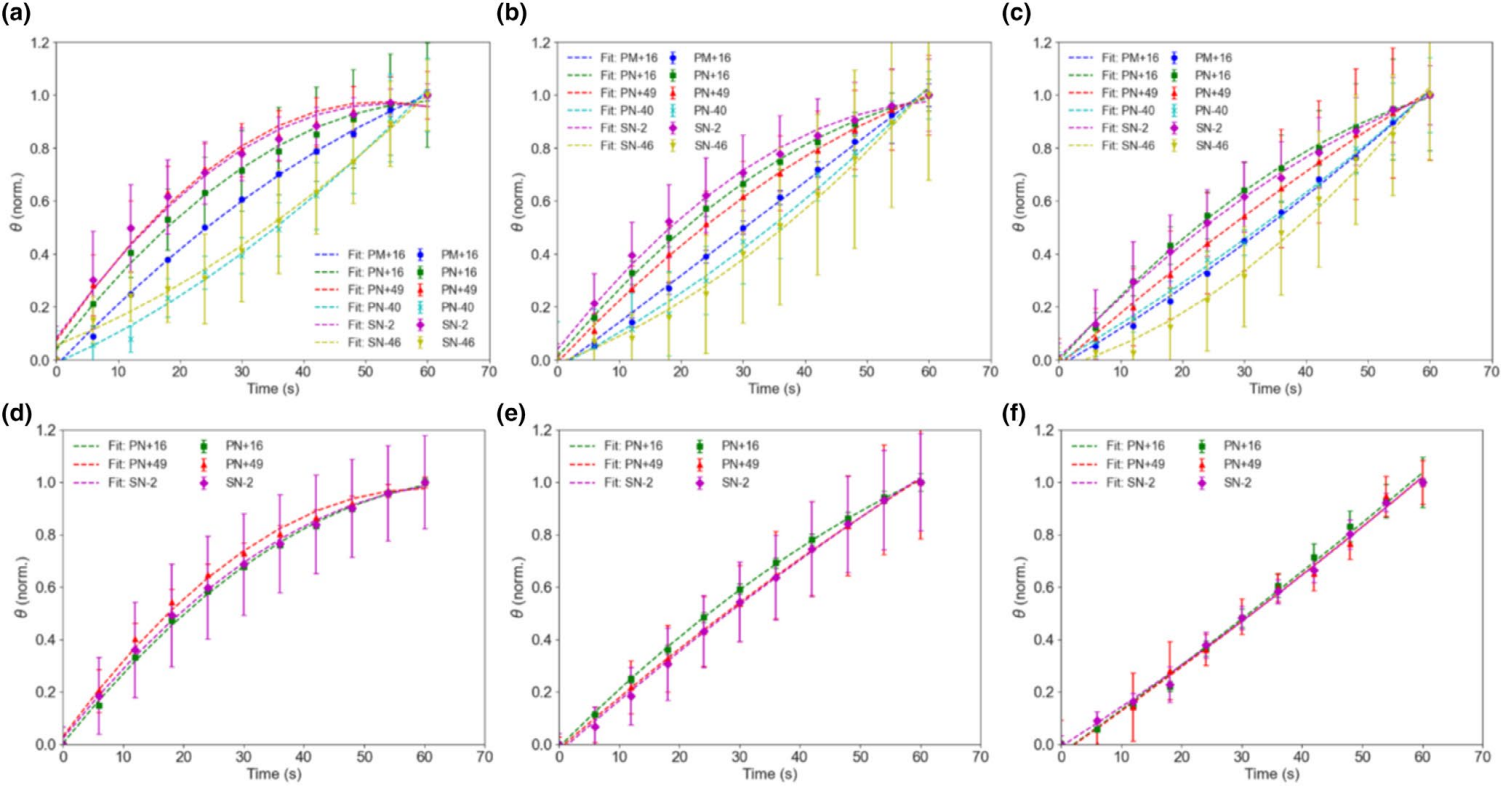
Normalized surface coverage (*θ*) over the first 60 s for healthy hair with (a) 0.01%, (b) 0.0025%, (c) 0.0005% w/v particle concentration added, and for damaged hair with (d) 0.01%, (e) 0.0025%, and (f) 0.0005% w/v particle concentration added. Fitting curves serves as a guide. [Colour figure can be viewed at wileyonlinelibrary.com]

As the particles deposited rather quickly on hair, the deposition kinetics were further analysed by focusing on the initial 60 s following particle addition (Figure 6). PN+49, PN+16, and SN‐2 exhibited quick deposition on healthy hair across all concentrations. However, this initial rate gradually decreased over time, most likely due to empty surface sites being taken up by particles [24]. This trend was further evident in the curve gradient in Equation (4), represented by coefficient *b*, for PN+49, PN+16, and SN‐2, whereby a higher *b* value meant a steeper initial gradient. The coefficient *b* also decreased with decreasing particle concentrations for these three particles (Table S2). In contrast, PN‐40, PM+16, and SN‐46 displayed a more linear deposition pattern over time, with lower *b* values. PN‐40 and SN‐46 consistently exhibited the lowest affinity, characterized by a positive coefficient *a* and low *b* values for all concentrations, indicating an upward polynomial curve with a gentle gradient. Their deposition rates remained relatively consistent at all concentrations, as previously observed. Similar to the observation over 2400 s after addition, PN+49 displayed a significant concentration‐dependent deposition rate, with lower affinity at lower concentrations and the highest rate at the highest particle concentration, as reflected in coefficients *a* and *b* (Table S2). Interestingly, PM+16 did not show any significant differences in deposition kinetics behaviour compared with the smaller‐sized particles, as its deposition was still more efficient than PN‐40 and SN‐46, though slower than PN+16.

For damaged hair, deposition rates were the fastest at the highest concentration for all three particles (PN+16, PN+49, and SN‐2), and decreased with time as empty sites got taken up (Figure 6d). At lower concentrations, the deposition curves exhibited a more linear behaviour (Figure 6e,f). The deposition rate for all three particles was also slower for damaged hair than healthy hair, as observed by a lower magnitude of coefficient *a* and *b* (Table S2). There was no observable difference between the deposition rate of the three particles as the curves overlapped, with only slight differences observable from the fitting coefficients. However, it is important to recognize that the differences in the curves, if any, are very slight, and careful interpretation is essential.

### SEM images

**FIGURE 7:**
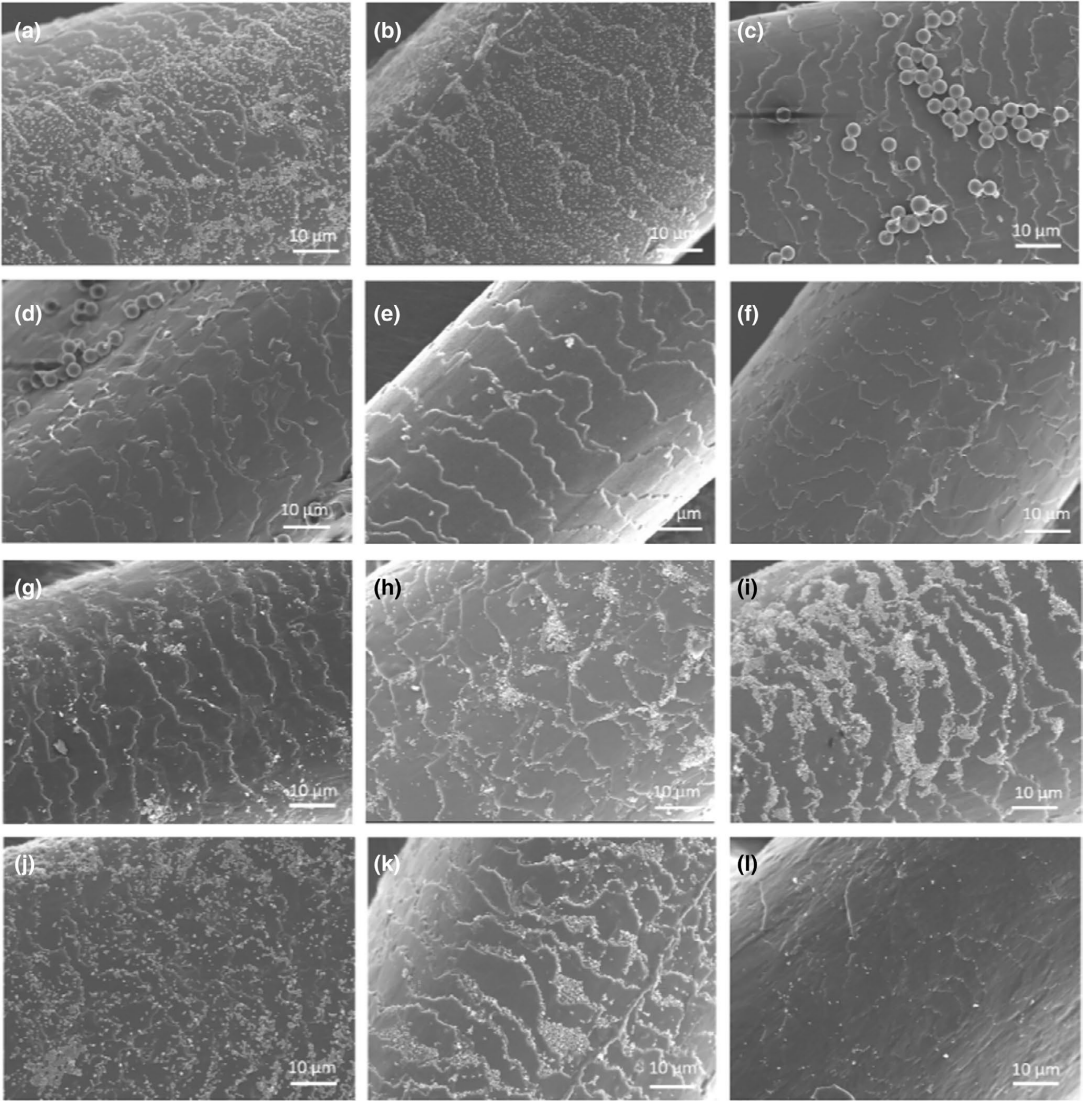
Representative SEM images of 0.01 w/v% concentration of PN+16 on (a) healthy hair and (b) damaged hair, PM+16 on (c) healthy and (d) damaged hair, PN‐40 on (e) healthy and (f) damaged hair, and PN+49 on (g) healthy and (h) damaged hair, SN‐2 on (i) healthy hair and (j) damaged hair, and of SN‐46 on (k) healthy and (l) damaged hair. Scale bars = 10 μm.

SEM imaging was performed on the hair tresses following particle adsorption at concentrations of 0.01%, 0.0025%, and 0.0005% w/v (Figure 7, Figures S1, S2). The SEM images were in good agreement with the surface coverage data obtained. Visually, the cuticles were all intact without exposed cortex. There were minimal PN‐40 particles observed on both healthy and damaged hair at all concentrations, while some PM+16 particles were visible on the hair surface. It appears that PN+16 covered the hair surface in a uniform manner, especially for damaged hair where no particle clumping was observed. Conversely, particle clumping was more apparent for PN+49 and SN‐2 on damaged hair. It was observed that SN‐2 and SN‐46 tended to aggregate along the cuticle edges, likely due to the exposed endocuticle, which is known to possess greater polarity as a result of protein exposure [25, 26]. This increased polarity likely promoted hydrogen bonding with the relatively hydrophilic SN‐2 and SN‐46. In contrast, PN+16, which is relatively more hydrophobic, accumulated less at the cuticle edges in comparison. Furthermore, the flat surface of healthy cuticles has a higher level of hydrophobicity, leading to a more noticeable contrast in hydrophobicity between the cuticle edge and the cuticle surface. This difference in hydrophobicities likely contributed to the localization of particles at the cuticle edges, which was more pronounced in healthy hair. In contrast, particles on damaged hair appeared to distribute more evenly. The same trend was observed for all particles at lower particle concentrations (Figures S1, S2) although fewer particles were observed.

Particle deposition is a complicated process with many factors at play and it is difficult to rank the influence of individual attraction forces. However, it is known that the resultant deposition is the net effect of attractive and repulsive forces. The particles were ranked according to their weighted surface coverage, and the factors affecting deposition are summarized semi‐quantitatively in Table 3.

**TABLE 3:** Summary of factors affecting deposition.

Particle	Rank		Factors affecting deposition	
	*θ*_healthy_	*θ*_damaged_	Healthy hair	Damaged hair
SN‐2	1	1	⚫⚫⚫⚪ Hydrogen bonding ⚫⚫⚫⚫ Low electrostatic repulsion	⚫⚫⚫⚫ Hydrogen bonding ⚫⚫⚫⚫ Low electrostatic repulsion ◉◉⚪⚪ Less binding sites
PN+16	2	2	⚫⚫⚪⚪ Hydrogen bonding ⚫⚫⚫⚫ Hydrophobic interactions ⚫⚫⚫⚪ Electrostatic attraction	⚫⚫⚫⚪ Hydrogen bonding ⚫⚫⚪⚪ Electrostatic attraction ◉◉⚪⚪ Less binding sites
PN+49	4	3	⚫⚫⚫⚪ Hydrophobic interactions ⚫⚫⚫⚫ Electrostatic attraction ◉◉◉⚪ Steric hindrance	⚫⚫⚪⚪ Electrostatic attraction ◉◉◉⚪ Steric hindrance ◉◉⚪⚪ Less binding sites
SN‐46	3	–	⚫⚫⚫⚪ Hydrogen bonding ◉◉◉◉ Electrostatic repulsion	⚫⚫⚫⚫ Hydrogen bonding ◉◉◉◉ Electrostatic repulsion ◉◉⚪⚪ Less binding sites
PM+16	5	–	⚫⚫⚪⚪ Hydrogen bonding ⚫⚫⚪⚪ Electrostatic attraction ◉◉◉◉ Low surface area to volume	⚫⚪⚪⚪ Hydrogen bonding ⚫⚪⚪⚪ Electrostatic attraction ◉◉◉◉ Low surface area to volume ◉◉⚪⚪ Less binding sites
PN‐40	6	–	⚫⚫⚪⚪ Hydrophobic interactions ◉◉◉◉ Electrostatic repulsion	◉◉◉◉ Electrostatic repulsion ◉◉⚪⚪ Less binding sites

*Note*: The factors affecting deposition are presented on a scale of 4; ⚫ represents factors which promote attraction and ◉ represents factors that inhibit attraction. Particles are ranked 1–6 in order of their weighted average deposition efficacy, 1 being the highest surface coverage, and 6 being the least surface coverage.

In this study, SN‐2 demonstrated the strongest affinity for both healthy and damaged hair. This can be attributed to its optimal level of hydrophilicity, allowing it to form hydrogen bonds with both the hair surface and the surrounding water. Moreover, the presence of amine groups on its surface contributed to its hydrophobicity, enabling the formation of hydrophobic interactions, while also aiding in charge neutralization. This neutralization minimized electrostatic repulsion from the hair, facilitating interaction. The presence of lone pair electrons on the amine group's nitrogen atom likely contributed to enhanced van der Waal's forces as well. Furthermore, the relatively small size of SN‐2's surface hydroxyl and amine groups had minimal steric hindrance. The similarity in charge between SN‐2 and the surface of damaged hair further heightened its affinity, possibly due to increased van der Waal's forces too. SN‐2's unexpected affinity for damaged hair suggests that having similar charges and the ability to form hydrogen bonds are important attractive forces, compensating for the limited interaction sites on damaged hair. SN‐2 was compared with SN‐46, which despite its high hydrophilicity and hence hydrogen‐bonding capacity, failed to deposit on damaged hair. This suggests that maintaining a low and similar charge as damaged hair may be the most crucial factor influencing affinity in this case.

It was interesting that PN+16 exhibited better deposition than SN‐2 at lower concentrations on healthy hair, with SN‐2 surpassing PN+16 only at higher concentrations. Similarly, PN+49 displayed rapid adsorption onto the hair surface even at low concentrations. This concentration‐dependent deposition phenomenon can be attributed to the long‐range effects of electrostatic attraction whereby at low concentrations when particles are farther away from the hair surface, electrostatic attraction still has influence. On the other hand, hydrogen bonding tends to operate within an intermediate range, so it becomes effective only when the particle concentration is sufficiently high for particles to approach the hair surface closely. This explains why particles like SN‐2, with its weaker electrostatic attraction than PN+16 and PN+49, only began to exhibit stronger adsorption at moderate to high particle concentrations.

The affinity of PN+16 and PN+49 to healthy hair was also much higher than PN‐40 which had a similar degree of wetting. This further highlights the prominent role of electrostatic attraction in deposition. However, PN+16 exhibited a notably greater affinity for healthy hair than PN+49, even though PN+49 possessed a higher positive charge than PN+16. This suggests that steric hindrance was effective in preventing deposition. Furthermore, for highly charged surfaces like PN+49 in electrolyte solutions, the influence of charge screening, as described by the Debye‐Hückel theory, tends to be substantial, resulting in a reduction of its effective charge [27].

Electrostatic attraction/repulsion is the most likely dominant force, followed by hydrogen bonding, and finally, hydrophobic interactions operate over the shortest range and become significant only when particles are in close proximity to the hair surface. This provides an explanation for why PN‐40 had the slowest adsorption onto the hair surface despite its high hydrophobicity, irrespective of particle concentration. It was also observed that larger particles (PM+16) deposited less compared with smaller particles, despite no significant differences in deposition kinetics between PM+16 and the nanoparticles. This suggests that the reduced deposition rate was influenced less by surface parameters and more by the effects of particle transport mechanisms. It should be noted that PM+16 is considered an inertial particle with trajectories that could deviate from the fluid motion and therefore could potentially have very different dominant deposition mechanisms, that is, particle transport mechanisms for larger particles are dominated by sedimentation or inertial impaction, whereas for smaller particles, diffusion and Brownian motion dominate [28, 29]. Intermolecular forces, such as electrostatic attraction, hydrogen bonding, and hydrophobic interactions tend to play a smaller role for larger particles too, for example, the small surface area to volume ratio of PM+16 reduced potential van der Waal's forces of attraction and hydration forces that could have otherwise promoted attraction.

This study followed the random sequential adsorption (RSA) model, which states that the maximum coverage of spherical particles on a surface is 54.7% [24]. The highest coverage obtained in this study was 15.3% (SN‐2 on damaged hair); however, SN‐2 and PN+16 had not reached equilibrium coverage even after 2400 s of interaction. By further increasing the deposition time, the surface coverage and hence particle affinity could be more accurately measured and compared. The particle concentration could also be increased till saturation to truly compare particle affinity. Nevertheless, the adsorption kinetics obtained was still able to demonstrate particle affinity to a great extent.

## CONCLUSIONS

This work explores the process of particle deposition onto hair using a streaming potential technique. Particle parameters, such as zeta potential, size, and hydrophobicity and their influence on deposition were considered. The findings lead to the conclusion that achieving the highest affinity for healthy hair involves striking a balance between having adequate hydrophilicity to foster hydrogen bonding while retaining a degree of hydrophobicity to facilitate hydrophobic interactions. Excessive negative charges or bulky surface functional groups should also be minimized to optimize affinity. This study also underscores the significance of hydrogen bonding and electrostatic attraction as predominant attraction forces, while the role of hydrophobic interactions appears less influential. Additionally, it was observed that highly charged particles are susceptible to charge screening effects, diminishing their effective charge with increasing distance from the particle.

To further explore how particle affinity is affected by hair damage, experiments were conducted on both healthy and damaged (bleached) hair. The results revealed that particles exhibit a greater deposition tendency on healthy hair compared with damaged hair, attributed to the diminished interaction sites due to the removal of the external lipid layer. SEM images also provided valuable insights into the preferred locations of particle deposition on the hair surface, shedding light on potential mechanisms influencing the effectiveness of hair products.

This study did not consider the impact of temperature and ionic strength of the solvent which are factors that can affect deposition and should be further investigated. Future research directions could also include consumer‐oriented aspects such as desorption studies and simulating real‐life hair product applications.

In conclusion, this study offers a foundational understanding of how particle attributes influence their affinity to hair and how hair damage levels impact particle deposition. Coupled with formulation expertise, the findings lay the groundwork for the development of more efficacious and long‐lasting hair care products.

## CONFLICT OF INTEREST STATEMENT

The authors declare that they have no known competing financial interests or personal relationships that could have influenced the work reported in this paper.

## Supporting information


Data S1:
