# Supplementary material for: Influence of particle parameters on deposition onto healthy and damaged human hair
Source: Int J Cosmet Sci. 2024 Aug 12;47(1):58–72. doi: 10.1111/ics.12994 (PMC11788003; doi:10.1111/ics.12994)
Supplement: Supplementary file 1 — Data S1: [file ICS-47-58-s001.docx]

**Supporting Information**

**Influence of Particle Parameters on Deposition onto Healthy and Damaged Human Hair**

Tham Huijun Phoebe^a^, Yip Kah Yuen^a^,_,_ Srinivasulu Aitipamula^b^, Srinivasa Reddy Mothe^b^, Zhao Wenguang^b^, Choong Ping Sen^b^, Ayca Altay Benetti^c^, Evonne Gan Wanjuan^d^, Fong Yew Leong^e^, Praveen Thoniyot^f^, Thomas Dawson^g,h *^

^a^ A*STAR Skin Research Labs (A*SRL), Agency for Science, Technology and Research (A*STAR), 11 Mandalay Rd, #17-01, Singapore 308232, Republic of Singapore

^b^ Institute of Sustainability for Chemicals, Energy and Environment (ISCE^2^), Agency for Science, Technology and Research (A*STAR), 1 Pesek Road, Jurong Island, Singapore 627833, Republic of Singapore

^c^ Department of Pharmacy, National University of Singapore, Singapore

^d^ School of Biological Sciences, Nanyang Technological University, 60 Nanyang Dr, Singapore 637551, Singapore

^e^ Institute of High Performance Computing (IHPC), Agency for Science, Technology and Research (A*STAR), 1 Fusionopolis Way, #16-16 Connexis, Singapore 138632, Republic of Singapore

^f^ Eindhoven University of Technology, Department of Chemical Engineering and Chemistry, Helix Building, PO Box 513, 5600 MB Eindhoven, The Netherlands

^g^ Center for Cell Death, Injury Regeneration, Departments of Drug Discovery Biomedical Sciences and Biochemistry Molecular Biology, Medical University of South Carolina, Charleston, SC, USA

^h^ A*STAR Skin Research Labs (A*SRL), Agency for Science, Technology and Research (A*STAR) & Skin Research Institute of Singapore (SRIS), 11 Mandalay Rd, #17-01, Singapore 308232, Republic of Singapore.

* corresponding author

**Table of Contents:**

Table S1: Table of R^2^ and initial gradient of logarithmic fitting curve for healthy and damaged hair over 2400s after particle addition.

Table S2: Table of coefficients for logarithmic fitting curve for healthy and damaged hair over 60s after particle addition.

Figure S1: Representative SEM images of 0.0025 w/v% concentration of particles on healthy hair and damaged hair.

Figure S2: Representative SEM images of 0.0005 w/v% concentration of particles on healthy hair and damaged hair.

| **Healthy hair** | | | | | | |
| --- | --- | --- | --- | --- | --- | --- |
| Conc Particle | 0.01% | | 0.0025% | | 0.0005% | |
|  | R^2^ | Gradient | R^2^ | Gradient | R^2^ | Gradient |
| PM+16 | 0.99 | 0.0024 | 1.00 | 0.0013 | 0.95 | 0.0020 |
| PN+16 | 0.98 | 0.0024 | 0.98 | 0.0021 | 0.94 | 0.0024 |
| PN+49 | 0.98 | 0.0035 | 0.99 | 0.0024 | 0.99 | 0.0019 |
| PN-40 | 0.98 | 0.0020 | 0.92 | 0.0009 | 0.96 | 0.0010 |
| SN-2 | 1.00 | 0.0020 | 0.98 | 0.0027 | 0.99 | 0.0021 |
| SN-46 | 0.95 | 0.0026 | 0.91 | 0.0028 | 0.98 | 0.0026 |
| **Damaged hair** | | | | | | |
| Conc Particle | 0.01% | | 0.0025% | | 0.0005% | |
|  | R^2^ | Gradient | R^2^ | Gradient | R^2^ | Gradient |
| PN+16 | 0.99 | 0.0021 | 1.00 | 0.0019 | 0.99 | 0.0021 |
| PN+49 | 0.98 | 0.0031 | 0.92 | 0.0025 | 0.92 | 0.0035 |
| SN-2 | 0.99 | 0.0018 | 0.98 | 0.0021 | 0.98 | 0.0019 |

Table S1: Table of R^2^ and initial gradient of logarithmic fitting curve for healthy and damaged hair over 2400s after particle addition.

| **Healthy hair** | | | | | | | | | | | | |
| --- | --- | --- | --- | --- | --- | --- | --- | --- | --- | --- | --- | --- |
| Conc Particle | 0.01% | | | | 0.0025% | | | | 0.0005% | | | |
|  | R^2^ | a | b | c | R^2^ | a | b | c | R^2^ | a | b | c |
| PM+16 | 0.998 | -1.1E-04 | 2.4E-02 | -7.3E-03 | 0.996 | 2.6E-05 | 1.6E-02 | -5.8E-03 | 0.997 | 8.2E-05 | 1.2E-02 | -2.5E-03 |
| PN+16 | 0.989 | -3.0E-04 | 3.5E-02 | -3.8E-02 | 0.997 | -2.2E-04 | 3.0E-02 | -2.8E-02 | 0.999 | -1.6E-04 | 2.6E-02 | -9.0E-03 |
| PN+49 | 0.976 | -4.0E-04 | 4.0E-02 | -2.9E-02 | 0.999 | -1.4E-04 | 2.5E-02 | -1.7E-02 | 0.789 | 3.4E-04 | 1.3E-04 | -8.6E-05 |
| PN-40 | 0.996 | 1.2E-04 | 9.8E-03 | -4.2E-03 | 0.993 | 1.1E-04 | 1.0E-02 | -1.6E-03 | 0.998 | 5.7E-05 | 1.4E-02 | -7.1E-03 |
| SN-2 | 0.971 | -3.9E-04 | 4.0E-02 | -2.8E-02 | 0.992 | -2.8E-04 | 3.3E-02 | -2.2E-02 | 0.998 | -1.5E-04 | 2.6E-02 | -2.1E-02 |
| SN-46 | 0.876 | 3.1E-04 | 1.5E-04 | -6.8E-05 | 0.959 | 3.1E-04 | 1.4E-04 | -7.6E-05 | 0.979 | 3.0E-04 | 1.6E-04 | -7.3E-05 |
| **Damaged hair** | | | | | | | | | | | | |
| Conc Particle | 0.01% | | | | 0.0025% | | | | 0.0005% | | | |
|  | R^2^ | a | b | c | R^2^ | a | b | c | R^2^ | a | b | c |
| PN+16 | 0.998 | -2.2E-04 | 3.0E-02 | -2.2E-02 | 1.000 | -1.0E-04 | 2.3E-02 | -1.3E-02 | 0.995 | 4.6E-05 | 1.5E-02 | -8.8E-03 |
| PN+49 | 0.992 | -3.1E-04 | 3.5E-02 | -3.1E-02 | 0.786 | 3.4E-04 | 1.2E-04 | -8.1E-05 | 0.991 | 5.4E-05 | 1.4E-02 | -1.9E-03 |
| SN-2 | 0.995 | -2.5E-04 | 3.2E-02 | -2.7E-02 | 0.806 | 3.4E-04 | 1.3E-04 | -8.4E-05 | 0.997 | 4.8E-05 | 1.4E-02 | -2.4E-03 |

Table S2: Table of coefficients for logarithmic fitting curve for healthy and damaged hair over 60s after particle addition.


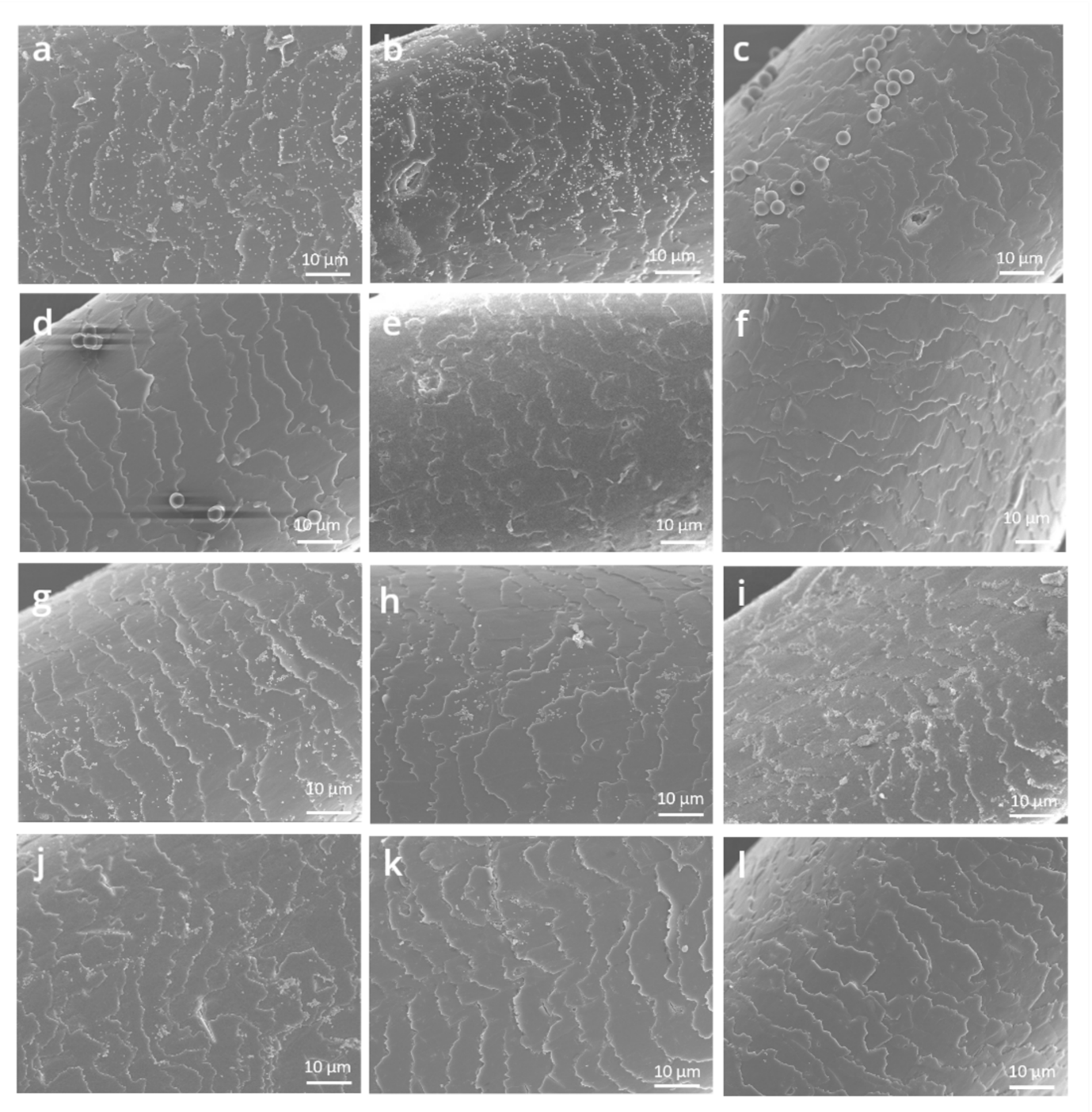


Figure S1: Representative SEM images of 0.0025 w/v% concentration of PN+16 on a) healthy hair and b) damaged hair, PM+16 on c) healthy and d) damaged hair, PN-40 on e) healthy and f) damaged hair, and PN+46 on g) healthy and h) damaged hair, SN-2 on a) healthy hair and b) damaged hair, and of SN-46 on c) healthy and d) damaged hair. Scale bars = 10 μm.


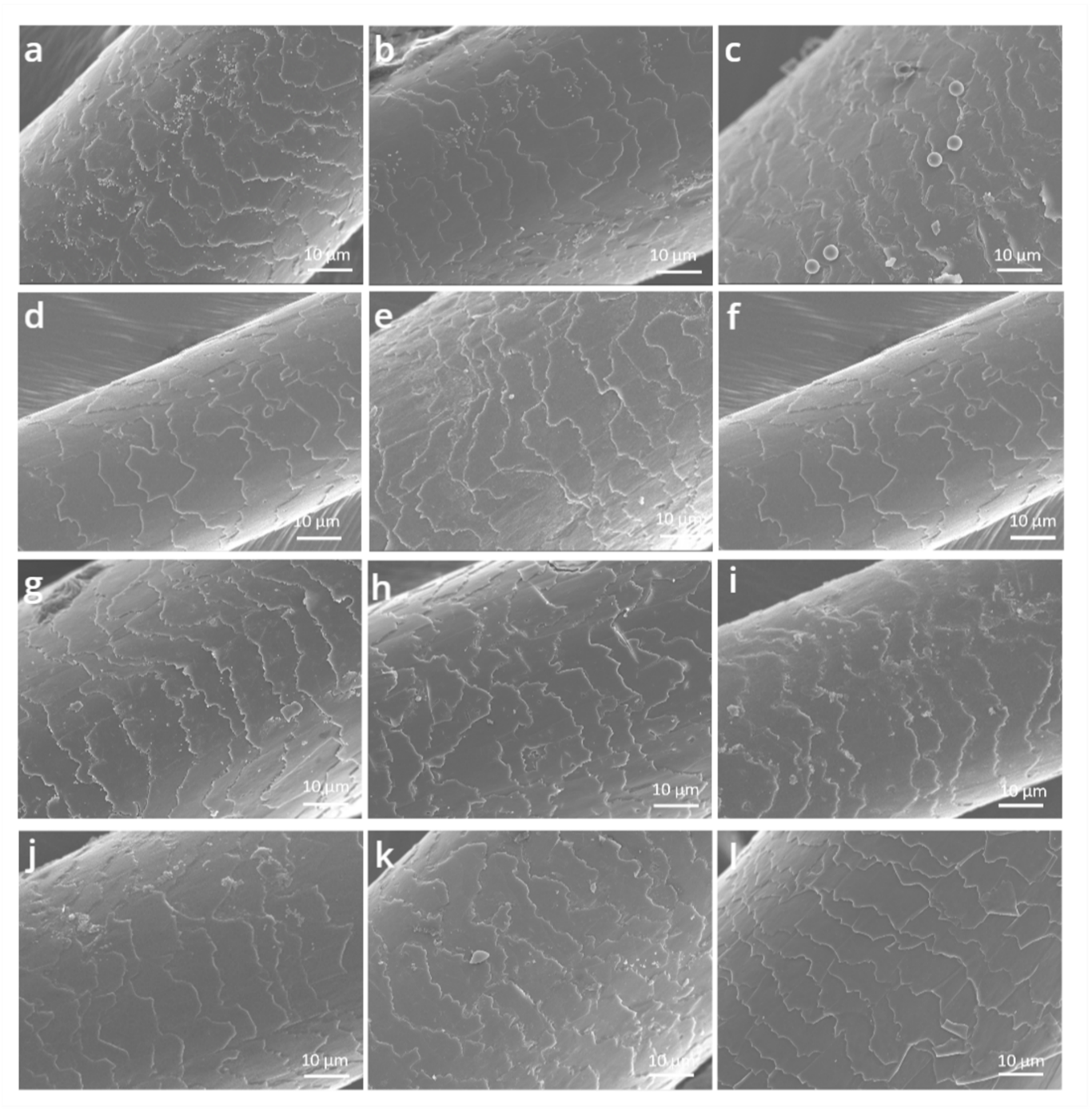


Figure S2: Representative SEM images of 0.0005 w/v% concentration of PN+16 on a) healthy hair and b) damaged hair, PM+16 on c) healthy and d) damaged hair, PN-40 on e) healthy and f) damaged hair, and PN+46 on g) healthy and h) damaged hair, SN-2 on a) healthy hair and b) damaged hair, and of SN-46 on c) healthy and d) damaged hair. Scale bars = 10 μm.
